# Study on Mechanical Properties of Composite Basalt Fiber 3D-Printed Concrete Based on 3D Meso-Structure

**DOI:** 10.3390/ma18143379

**Published:** 2025-07-18

**Authors:** Shengxuan Ding, Jiren Li, Mingqiang Wang

**Affiliations:** School of Civil Engineering, University of Science and Technology Liaoning, Anshan 114051, China; 24210859010812@ustl.edu.cn (S.D.); 24210859010838@stu.ustl.edu.cn (M.W.)

**Keywords:** 3D printing concrete, three-dimensional mesoscopic, anisotropy, numerical simulation, failure mechanism

## Abstract

As 3D concrete printing emerges as a transformative construction method, its structural safety remains hindered by unresolved issues of mechanical anisotropy and interlayer defects. To address this, we systematically investigate the failure mechanisms and mechanical performance of basalt fiber-reinforced 3D-printed magnesite concrete. A total of 30 cube specimens (50 mm × 50 mm × 50 mm)—comprising three types (Corner, Stripe, and R-a-p)—were fabricated and tested under compressive and splitting tensile loading along three orthogonal directions using a 2000 kN electro-hydraulic testing machine. The results indicate that 3D-printed concrete exhibits significantly lower strength than cast-in-place concrete, which is attributed to weak interfacial bonds and interlayer pores. Notably, the R-a-p specimen’s Z-direction compressive strength is 38.7% lower than its Y-direction counterpart. To complement the mechanical tests, DIC, CT scanning, and SEM analyses were conducted to explore crack development, internal defect morphology, and microstructure. A finite element model based on the experimental data successfully reproduced the observed failure processes. This study not only enhances our understanding of anisotropic behavior in 3D-printed concrete but also offers practical insights for print-path optimization and sustainable structural design.

## 1. Introduction

In recent years, frequent and devastating earthquakes in various parts of the world have highlighted critical shortcomings in traditional cast-in-place concrete structures, particularly their vulnerability to brittle failure and poor interfacial bonding under dynamic loading [1,2,3]. Structural collapses due to inadequate material strength, uneven curing, and construction quality defects continue to result in substantial human and economic losses. These challenges emphasize the urgent need for novel construction materials and methods that offer greater consistency, anisotropic control, and enhanced durability under multi-axial stress conditions [4,5,6]. With the global construction industry’s urgent demand for efficient, personalized, and sustainable construction methods, 3D printed concrete (3DPC) technology has emerged and advanced rapidly, leveraging the unique advantages of layer-by-layer deposition. This technology enables the efficient fabrication of complex structures, significantly shortening construction timelines, reducing labor requirements, and demonstrating substantial potential for minimizing material consumption and construction waste generation. Consequently, 3DPC presents significant opportunities for innovation within the construction sector [7,8,9].

However, the core challenge of 3DPC is its inherent anisotropic mechanical behavior, which originates from the layer-by-layer deposition process during printing [10,11]. The interlayer interface region typically constitutes a weak zone, resulting in significantly lower tensile strength, shear strength, and overall structural integrity compared to traditional cast-in-place concrete [12,13,14]. Studies have demonstrated that microstructural defects at the interface, such as pores and inadequate hydration, constitute the primary factor governing crack initiation and propagation [15]. To address this performance limitation, material modification strategies are being actively pursued. These include the incorporation of basalt fiber (BF), leveraging its high tensile strength, corrosion resistance, and excellent matrix compatibility [16]. Xiao et al. [17] demonstrated that basalt fiber (BF) can enhance compressive strength along specific directions (e.g., the X-direction) by 15–20%. However, strength improvement in the vertical direction (Z-direction) remains constrained by fiber distribution limitations. Furthermore, as a critical manufacturing process parameter, the printing path exerts a predominant influence on the mechanical properties of the final component. Different printing paths, such as the commonly used strip path and contour path (or loop path), result in distinct interfacial morphologies and distributions [18]. Experiments by Le et al. [19] demonstrated significant mechanical anisotropy in specimens formed using the strip path. Specifically, the strength parallel to the printing direction (X-direction) was the highest, while the strength perpendicular to the strip direction (Y-direction) and the interlayer direction (Z-direction) were comparable but low. The numerical investigation by Xiao et al. [17] further established that interfacial slip between adjacent printed strips constitutes the primary mechanism responsible for the reduced strength of 3DPC compared to cast-in-place concrete. Regarding numerical simulation, Bi et al. [20] developed a topology optimization framework accounting for anisotropy; however, their work did not validate the framework for hybrid path designs. Furthermore, Liu et al. [21] elucidated the influence of pores on mechanical properties through CT scanning and simulation. Nevertheless, they did not comprehensively investigate the complex coupling effects among printing path spacing, fiber orientation, and pore distribution. This limitation constrains the model’s predictive capability under complex conditions.

Current research on the influence of printing paths primarily focuses on single-path modes, such as the strip or contour path. However, the systematic exploration of more complex composite path designs, which may enhance performance, is notably lacking [22]. Furthermore, the multi-factor coupling mechanism among printing path parameters, fiber orientation distribution, and interfacial micro-defects—including pore distribution and hydration state—remains unclarified. How these factors collectively govern the material’s anisotropic behavior and ultimate failure mechanism has yet to be comprehensively elucidated and quantified [23]. Existing numerical models exhibit inherent limitations in comprehensively integrating path-induced fiber orientation, microstructural defects, and their complex interactions. Consequently, this impedes the accurate prediction of the mechanical response in components produced using complex printing paths.

To address these knowledge gaps, this study aims to systematically investigate the anisotropic mechanical behavior and failure mechanisms of 3D-printed basalt fiber-reinforced concrete, focusing on the coupled effects of printing path geometry, fiber orientation, and pore distribution. Specimens were fabricated using advanced robotic extrusion systems with composite path designs to simulate real-world engineering conditions. Subsequently, compressive and splitting tensile tests were conducted along three orthogonal directions. To elucidate the micro–macro-mechanical relationships, Digital Image Correlation (DIC), X-ray computed tomography (CT), and scanning electron microscopy (SEM) were employed. Furthermore, a three-dimensional mesoscale finite element model incorporating interfacial weakening effects was developed and validated against experimental results.

The novelty of this study lies in its comprehensive integration of printing process parameters, fiber-induced anisotropy, and microstructural defects into a unified modeling and experimental framework. This work provides a theoretical and practical basis for optimizing 3D concrete-printing parameters, improving material reliability, and promoting the broader application of sustainable digital construction technologies.

## 2. Experiment

### 2.1. Material

**Figure 1:**
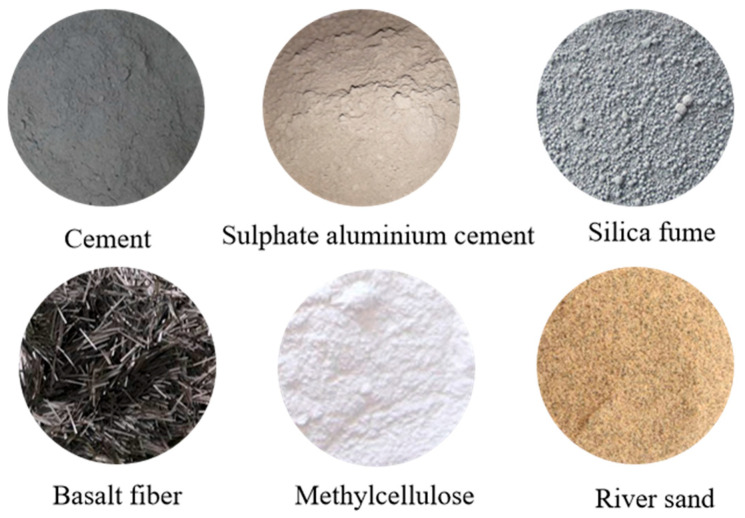
Raw materials.

The composition of the raw materials used in this study is as follows: Cement was obtained from Shenyang Zhuonengda Special Materials Co., Ltd. (Shenyang, China). The fine aggregates and river sand were taken from Shandong Weifang Fuzhu Building Materials Co., Ltd. (Weifang, China) and Liaoning Anshan, respectively. Silica fume, superplasticizer, and hydroxypropyl methyl cellulose were provided by Tianjin Zhiyuan Chemical Reagent Co., Ltd. (Tianjin, China), while basalt fiber was from Jiangsu Yancheng Haining Anjie Composite Materials Co., Ltd. (Yancheng, China). The specific information on raw materials is shown in Figure 1. The cement (OPC) comprises P.O 42.5-grade ordinary Portland cement and Type 52.5 rapid-hardening sulphoaluminate cement (SAC). Fine aggregates (FAs) consist of natural river sand with a particle size range of 40 to 70 mesh and a maximum particle size not exceeding 0.35 mm. Silica fume (SF) has a silica (SiO_2_) content of 94% and a loss on ignition (LOI) of 1.5%. Mixing water (W) is tap water. Hydroxypropyl methyl cellulose (HPMC) is a white powder with a viscosity of 100,000 mPa·s and a density of 1.41 g/cm^3^. The mix proportions (by mass) of the 3D printable concrete materials are listed in Table 1.

The material composition was determined based on a constructability assessment following the relevant national standard [24]. This guideline provides a systematic evaluation framework for 3D printable concrete, covering key indices such as extrudability, buildability, and structural stability. The selected mix satisfies the performance requirements specified in the standard, demonstrating continuous filament extrusion, interlayer adhesion, and shape retention under the layer-by-layer deposition process. These criteria collectively ensure that the mixture is suitable for 3D printing construction applications.

The materials selected in this study are commonly used in 3D-printed concrete applications. Sulphoaluminate cement is particularly favored for its rapid setting, early strength development, and excellent printability, which ensures layer stability during printing without the need for formwork. Basalt fiber is also widely adopted in the field of additive construction due to its high tensile strength, corrosion resistance, and excellent compatibility with cementitious matrices. The synergy between SAC and basalt fiber not only improves the printability and dimensional stability of fresh concrete but also enhances the toughness and cracking resistance of hardened components.

The innovation of this work lies in the combination of these two materials within a mesostructure-aware framework, enabling detailed investigation into the anisotropic mechanical behavior under different printing paths and loading orientations. This approach provides a robust foundation for optimizing material design in complex-geometry and high-stress 3D-printed structural elements.

### 2.2. Specimen Design

The basalt fiber-reinforced 3D printable concrete was prepared as follows: First, P.O 42.5-grade ordinary Portland cement and silica fume (with SiO_2_ content of at least 94%) were dry-mixed at a low speed for 3 min. Next, a polycarboxylate superplasticizer solution was prepared and gradually incorporated into the dry mixture, followed by medium-speed mixing for another 3 min to form a uniform slurry. Then, Type 52.5 rapid-hardening sulphoaluminate cement and natural river sand (40–70 mesh) were sequentially added, with mixing continued for 3 min. Hydroxypropyl methyl cellulose (HPMC, viscosity 100,000 mPa·s) was then sprinkled into the mixture and blended at low speeds for 1 min, after which basalt fibers were incrementally added and mixed for an additional 3 min to ensure a homogeneous and workable slurry. The slurry was loaded into the printing hopper, and specimens were printed according to predefined parameters, with the interlayer time interval strictly controlled to within 20 min. After printing, the specimens were stored undisturbed for 24 h. They then underwent 2 days of hot water bath curing at 90 ± 2 °C, followed by transfer to a standard curing room (20 ± 2 °C, RH > 95%) for 28 days. Finally, the cured specimens were cut to the required dimensions and surface-polished.

The desktop 3D concrete printing system has an overall dimension of 1370 mm × 1170 mm × 1460 mm, with a build volume of 600 mm × 600 mm × 550 mm. It operates at a print speed of 40 mm/s and an extrusion rate of 60 cm^3^/s, utilizing a circular nozzle with a 20 mm outlet diameter.

For sample fabrication, a contour-parallel toolpath was applied to the outer perimeter, while the internal region was infilled using a rectilinear pattern. The deposited filament had a width of 25 mm and a layer height of 20 mm. Printed specimens measured 150 mm × 100 mm × 100 mm.

The formulated basalt fiber-reinforced 3D printable concrete exhibited excellent printability with no observable slump deformation.

### 2.3. Specimen Preparation and Loading

**Figure 2:**
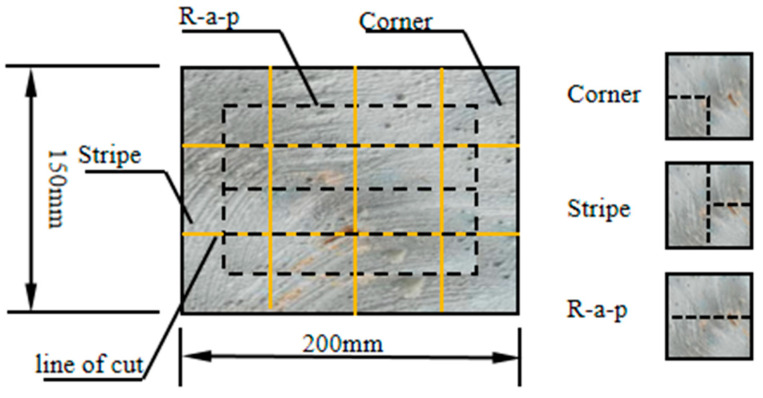
Three-dimensional printed concrete specimen cutting.

**Figure 3:**
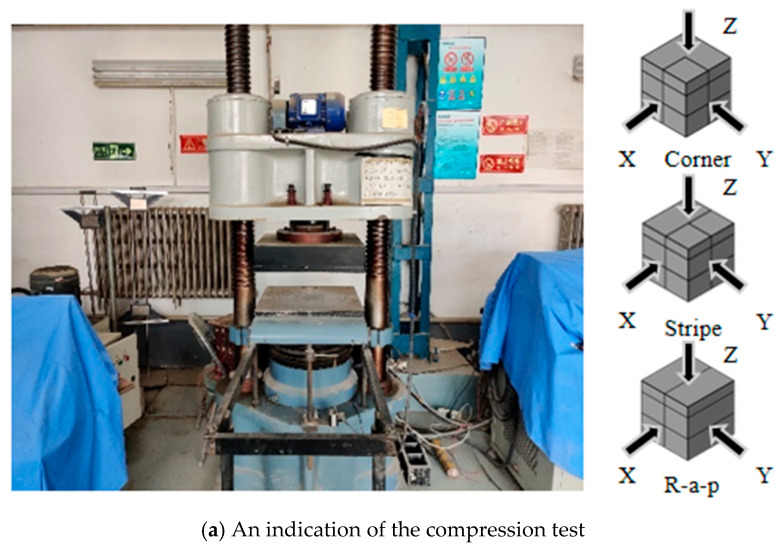

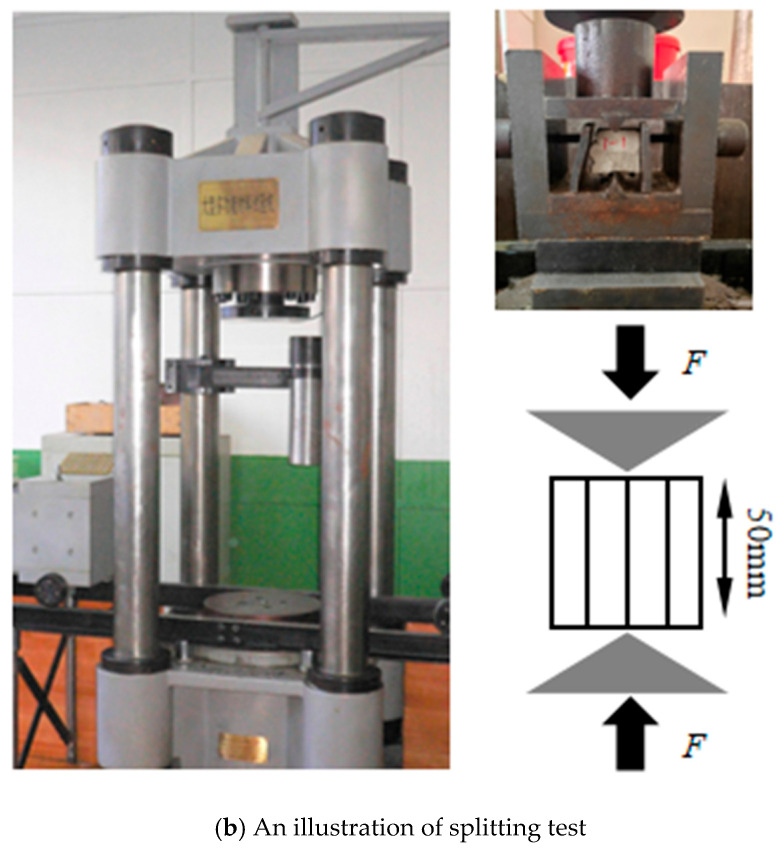
Three-dimensional printed concrete mechanical test schematic diagram.

A 2000 kN electro-servo compression testing machine was employed to apply quasi-static loading to the composite 3D-printed concrete specimens. Testing was conducted in accordance with references [24,25] at a constant loading rate of 0.002 m/s^2^. Following the sectioning of each plate specimen, three types of test coupons were obtained, Corner, Stripe, and R-a-p, as illustrated in Figure 2. In this test, three types of specimens were prepared: Corner cubes were cut from the four corners of the sheet. Stripe cubes were obtained from the side edge of the plate (strip-shaped region). R-a-p (Radial Position) cubes were taken from the mid-section of the plate (radial direction region). For each specimen type, the 28-day compressive strength and splitting tensile strength were measured on 50 mm side length cubes. Testing was conducted along three orthogonal directions for each specimen type, as illustrated in Figure 3. Five parallel specimens were tested for each combination of specimen type and test (compressive or tensile). To investigate the failure mechanisms under different compressive and splitting tensile loading directions, the interfaces were categorized into two types based on their relationship to the printing process: interlayer interfaces and inter-bead interfaces:

Interlayer Interface: The interface between vertically adjacent deposited layers, primarily subjected to the self-weight load from the concrete above.

Inter-bead Interface: The bonding interface between adjacent extrusion beads within the same layer, primarily subjected to lateral compressive stresses during printing.

Research indicates that incorporating basalt fiber (BF) results in a slight enhancement in the compressive strength of concrete while significantly improving its splitting tensile strength and enhancing overall toughness.

### 2.4. DIC Test of 3D Printed Concrete

After curing for 28 days, compression specimens were tested using Digital Image Correlation (DIC), a technique that quantitatively characterizes failure modes and maps the full-field strain and displacement distributions within cube specimens [26]. The experimental setup included a camera with a resolution of 3376 × 2499 pixels, capturing images at a rate of five per second during loading, and the testing protocol followed the Software Reference Manual for Vic-3D v8.

### 2.5. Three-Dimensional Printed Concrete CT Scan

Industrial X-ray computed tomography (CT) scanning was performed using a multi-scale voxel system. Following 28 days of curing, Corner specimens (50 mm cubes) were scanned at an isotropic voxel resolution of 102.713 μm. The scanning parameters utilized a 450 kV X-ray source with current settings of 1.60 mA and 3.30 mA. Each specimen was reconstructed from 1000 radiographic projections, yielding a 3072 × 3072 pixel matrix covering a 427 mm × 427 mm field of view. Data denoising was performed using a median filter. To ensure data validity, slice images corresponding to the axial extremities of the cubical specimen were cropped. Following 28-day curing, CT-scanned data underwent preliminary reconstruction using VoxelStudioRecon 3D image reconstruction software (version 2.1.0, Thermo Fisher Scientific, Waltham, MA, USA) to generate initial tomographic projections [27]. Subsequently, Avizo software(version 9.5.0, Thermo Fisher Scientific, Waltham, MA, USA) was employed for data preprocessing to quantify microstructural parameters, including the porosity, pore size distribution, pore volume, pore morphology, and centroid coordinates of pores.

### 2.6. Micro-Test of 3D Printed Concrete

Microstructural analysis was conducted using high-resolution scanning electron microscopy (SEM) on specimens cured for 28 days. Samples were extracted from 50 mm 3D-printed concrete cubes, which were prepared with flat, representative surfaces for SEM imaging. Specimens were sectioned and polished to achieve SEM-compatible dimensions with representative, artifact-free surfaces. At a typical field width of 30 μm, surfaces were scanned under controlled electron beam conditions (accelerating voltage of 5.0 kV and working distance of 11 mm) calibrated for optimal surface interaction. This configuration enabled sub-surface penetration for micro-morphological characterization. Multiple high-magnification images were systematically acquired to document microstructural features.

### 2.7. Establishment of Numerical 3D Printed Concrete Model

In order to explore the effects of extrusion-induced porosity and anisotropy in 3D printed concrete, a three-dimensional porous media model was constructed using a Monte Carlo random sampling method. The concrete domain was first discretized into a grid, and random coordinates were generated to place spherical voids representing pores, which were then deleted to simulate material discontinuities [28,29]. The base substrate and the interlayer gap regions were superimposed to form the complete specimen geometry.

**Figure 4:**
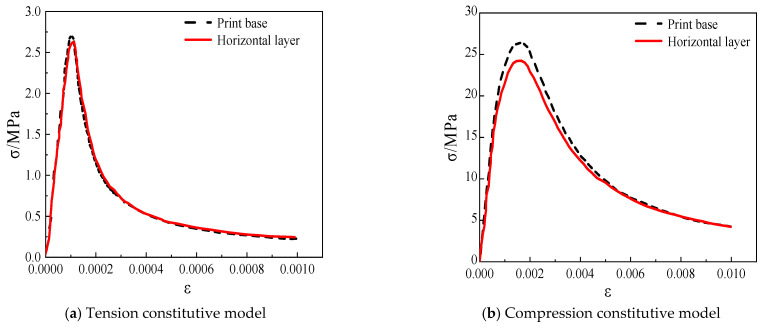
Constitutive model of 3D-printed concrete material.

To simulate the nonlinear mechanical response of the material, a Concrete Damaged Plasticity (CDP) model was adopted as the constitutive framework [29,30], with the 3DPC CDP constitutive data provided in Table 2. This model integrates plastic deformation and isotropic damage evolution. The plastic yield and failure surfaces were defined using the Drucker–Prager criterion, with calibration performed based on peak stress and strain values from experimental data, as shown in Figure 4.

In the softening stage, a linear damage evolution law was applied for both tension and compression. The elastic stiffness degraded progressively according to a scalar damage variable *d* ∈ [0, 1], where *C* is the undamaged elastic stiffness tensor.

Finite element analysis was performed in numerical simulation software/Explicit (https://www.3ds.com/products/simulia/abaqus/explicit, accessed on 20 June 2025), with the CDP model implemented via a user-defined subroutine (VUMAT). A dynamic explicit scheme was used to capture crack propagation and brittle failure under both compressive and splitting tensile loading. The steel loading plates in the splitting test were modeled with surface-to-surface contact: “hard” contact was applied in the normal direction, and Coulomb friction was adopted in the tangential direction. The frictional shear stress was calculated using *τ*_crit_ = *μp*, where *μ* = 0.6 is the friction coefficient and *p* is the contact pressure. The steel plates were set as master surfaces, and the concrete blocks was set as slave surfaces.

**Figure 5:**
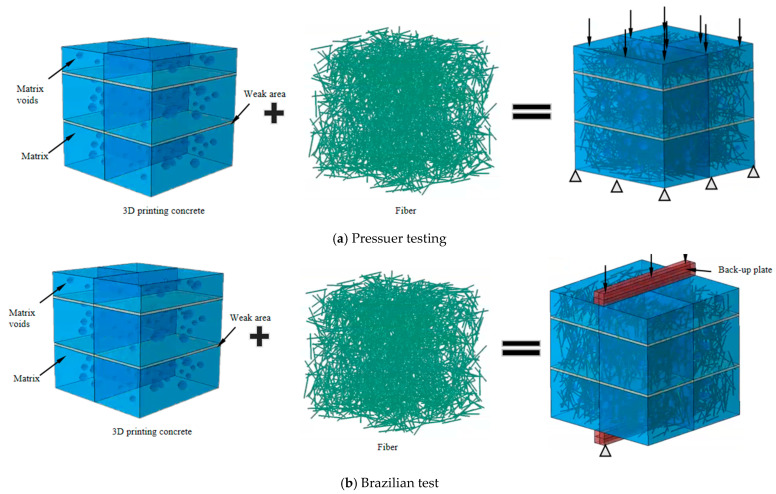
Numerical model of the Corner test block.

Furthermore, basalt fibers were modeled as embedded inclusions within the concrete matrix using the Embedded Region technique in numerical simulation software. This allowed internal interaction between the fiber phase and concrete without requiring explicit contact definitions. Taking the Corner specimen as an example, a uniform compressive load was applied to the top end plate, and the bottom surface was fixed in all degrees of freedom, as shown in Figure 5.

## 3. Results and Discussions

### 3.1. Compression Test Results and Analysis

Compressive failure in 3D-printed concrete exhibits significant anisotropy. Under X-direction loading, cracks propagate along fiber–matrix debonding paths, forming gridded through-thickness fracture networks. Y-direction compression failures are primarily governed by interlayer interfaces, manifesting as stepwise or wavy crack patterns. Basalt fiber bridging effectively impedes crack coalescence [16]. The fibers’ high tensile strength promotes distributed microcracking failure modes, while their low elastic modulus provides limited restraint against transverse expansion. At higher dosage rates, frictional interlocking enhances interlayer shear strength, exhibiting a brittle-to-ductile transition in failure behavior. The matrix’s strength is higher than that of ordinary concrete, and the brittleness is obvious at the initial stage of failure, and the residual strength is higher due to the fiber pull-out effect at the later stage. The Z-direction compression failure is dominated by cones. The self-weight compaction of the printing layer and the vertical continuous distribution of the fibers make its behavior close to that observed in cast-in-place concrete. The cracks form 45° oblique cracks, and the fiber bridging causes crack bifurcation to increase. Interlayer weakening has an implicit effect on the failure path. The poor control of the printing layer’s thickness may cause secondary longitudinal cracks along the layer boundary. The alkali resistance of basalt fiber can inhibit interfacial chemical degradation, but insufficient physical bonding will lead to interlayer slip microcracks [31]. Inter-bead pores act as stress concentrators in the X and Y directions, where fiber rigidity may exacerbate microcrack nucleation at pore peripheries. Extrusion-induced horizontal fiber alignment enhances the mechanical contribution efficiency in the Z-direction relative to X/Y orientations. The pronounced bearing capacity’s anisotropy in 3D-printed concrete specimens stems primarily from printing-induced interlayer weakening and microstructural heterogeneity. Quantitative analyses reveal the following: R-a-p specimens exhibit 38.7% lower Z-direction capacity versus the Y-orientation. Corner specimens show 7.87% reduced X-direction capacity compared to the Y-orientation.

**Figure 6:**
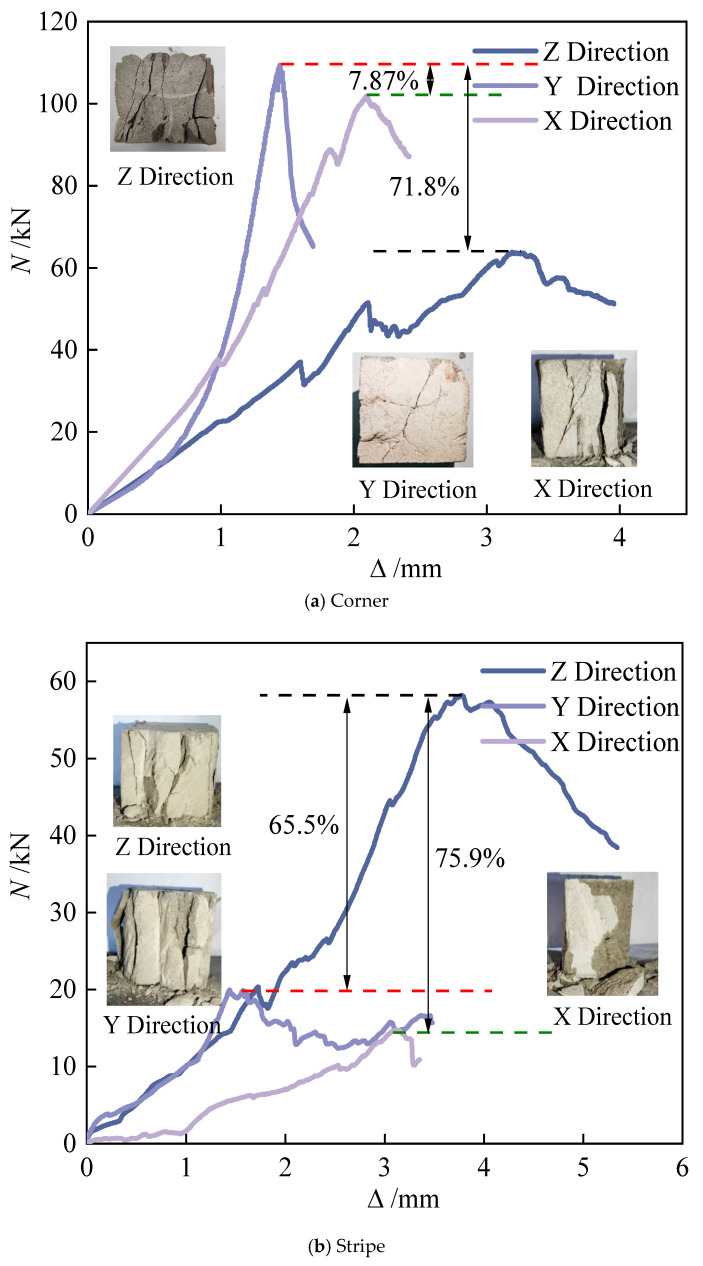

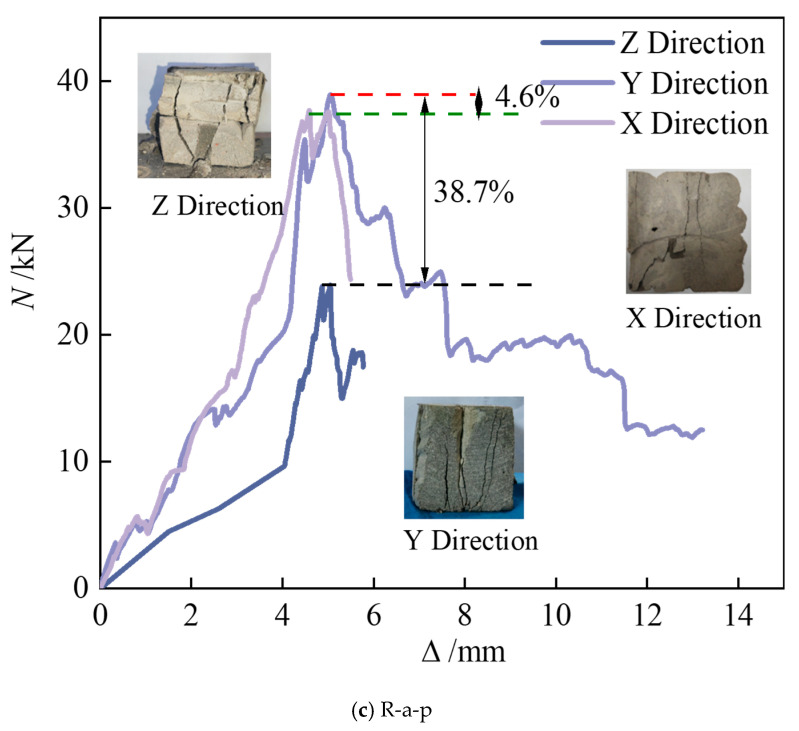
Load–displacement curves of the compressive test block under different loading directions.

Stripe specimens demonstrate significantly enhanced Z-direction performance, indicating that strategic deposition path designs can reconfigure mechanical advantages through controlled material stacking patterns. All specimen types exhibit consistent load–displacement behavior across loading directions (Figure 6): rapid initial displacement progression, moderated mid-stage deformation, and terminal brittle fracture.

Compressive strength variations exist across printed specimen types. Corner specimens exhibit the highest Y-direction strength (*f*_y_ = 43.6 MPa), exceeding Stripe’s *f*_z_ and R-a-p’s *f*_y_ by 46.78% and 64.2%, respectively. Their tri-directional mean strength (36.8 MPa) also demonstrates superiority, surpassing Stripe and R-a-p specimens by 66.6% and 64.1%, respectively. This enhancement stems from the confinement effect: Initially printed perimeter concrete radially constrains subsequently deposited material, improving compaction in striped regions. Conversely, R-a-p specimens yield the lowest mean strength due to extensive weak interfacial transition zones formed during layer-by-layer deposition. Notably, X- and Y-direction strengths remain comparable across all specimen types, which is attributable to identical interfacial bonding characteristics on these loading planes.

**Figure 7:**
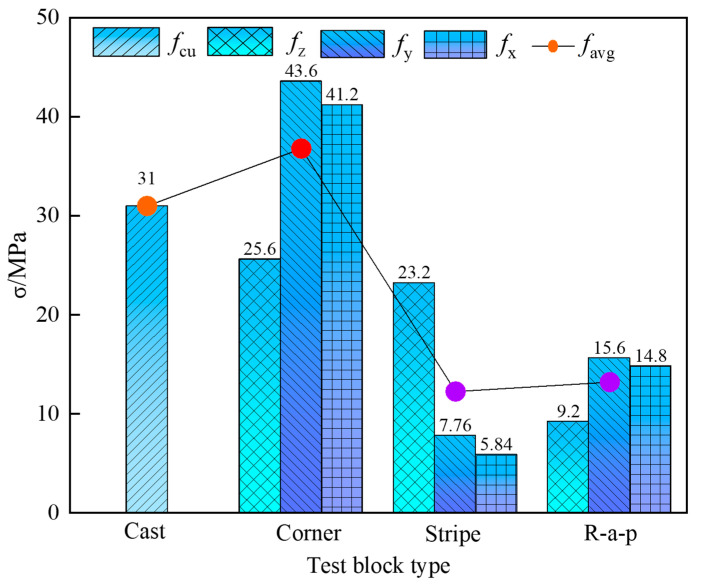
The compressive strength of three kinds of printed test blocks in each direction and the cast-in-place test block. Comparison of compressive strength.

Figure 7 compares the directional compressive strengths (*f*_x_, *f*_y_, and *f*_z_) of three printed specimen types against the compressive strength (*f*_cu_) of cast-in-place concrete. All printed specimens exhibit lower strength values in every orientation compared to their cast-in-place counterparts under identical conditions, as shown in Table 3. This reduction is primarily attributed to weak interlayer and inter-bead interfaces within printed specimens, which promote stress concentration and precipitate premature crack initiation. The anisotropy coefficient *I*a [19] was employed to evaluate the compressive strength anisotropy of the three types of test blocks, as calculated using Equation (1).

(1) $$I_{a}=\sqrt{{\left(f_{x}-f_{avg}\right)}^{2}+{\left(f_{y}-f_{avg}\right)}^{2}+{\left(f_{z}-f_{avg}\right)}^{2}}$$

In the above formula, *f*_avg_ represents the average compressive strength across the three loading directions. The anisotropy coefficients for the Corner test block, Stripe test block, and R-a-p test block are 17.08, 35.12, and 31.22, respectively. This indicates that the Stripe test block exhibits the most pronounced anisotropy, while the Corner test block shows the least pronounced anisotropy.

### 3.2. Results and Analysis of Splitting Test

Basalt fiber-reinforced 3D-printed concrete exhibits distinct directional failure modes under splitting tension, governed by anisotropic interlayer interface strength, extrusion bead geometry, and porosity distribution:

X-direction Loading: Primary crack propagation along interlayer interfaces generates planar fracture surfaces, manifesting interfacial delamination failure.

Y-direction Loading: Crack advancement through inter-bead discontinuities produces stepwise fracture morphology, characteristic of localized step-failure.

Z-direction Loading: Vertical crack propagation along deposition planes creates smooth separation surfaces, indicating transverse layer failure.

In contrast, cast-in-place concrete monoliths exhibit homogeneous failure patterns with isotropic crack propagation. Fracture paths develop uniformly along maximum principal stress trajectories, forming continuous rupture planes without directional preference. The fiber bridging effect of basalt fibers in 3D-printed concrete exhibits directional dependency due to constraints imposed by interlayer interfaces and geometric discontinuities. In contrast, cast-in-place concrete demonstrates uniform fiber distribution with pronounced bridging effectiveness [32]. Consequently, the fracture behavior of 3D-printed concrete is predominantly governed by deposition parameters, manifesting anisotropic failure characteristics. Cast-in-place specimens exhibit monolithic failure patterns dictated by material homogeneity and bulk strength.

**Figure 8:**
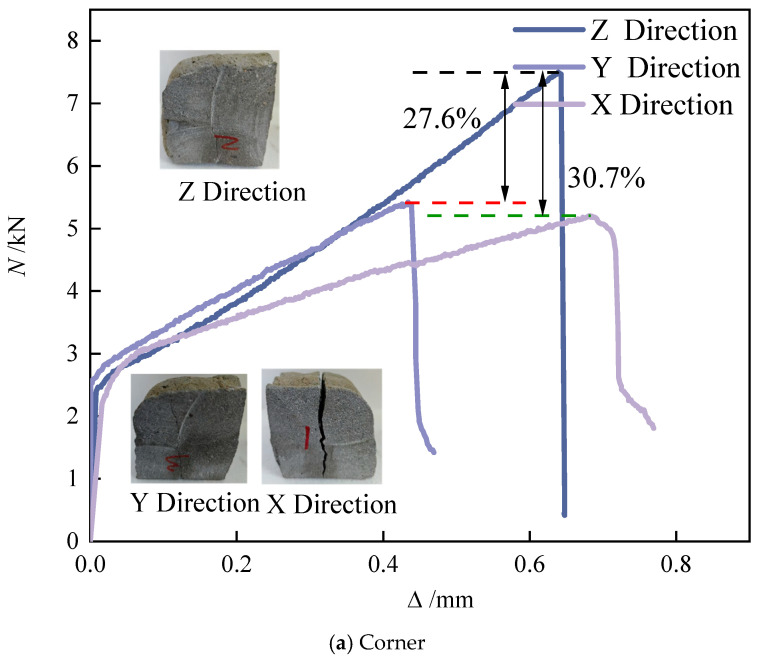

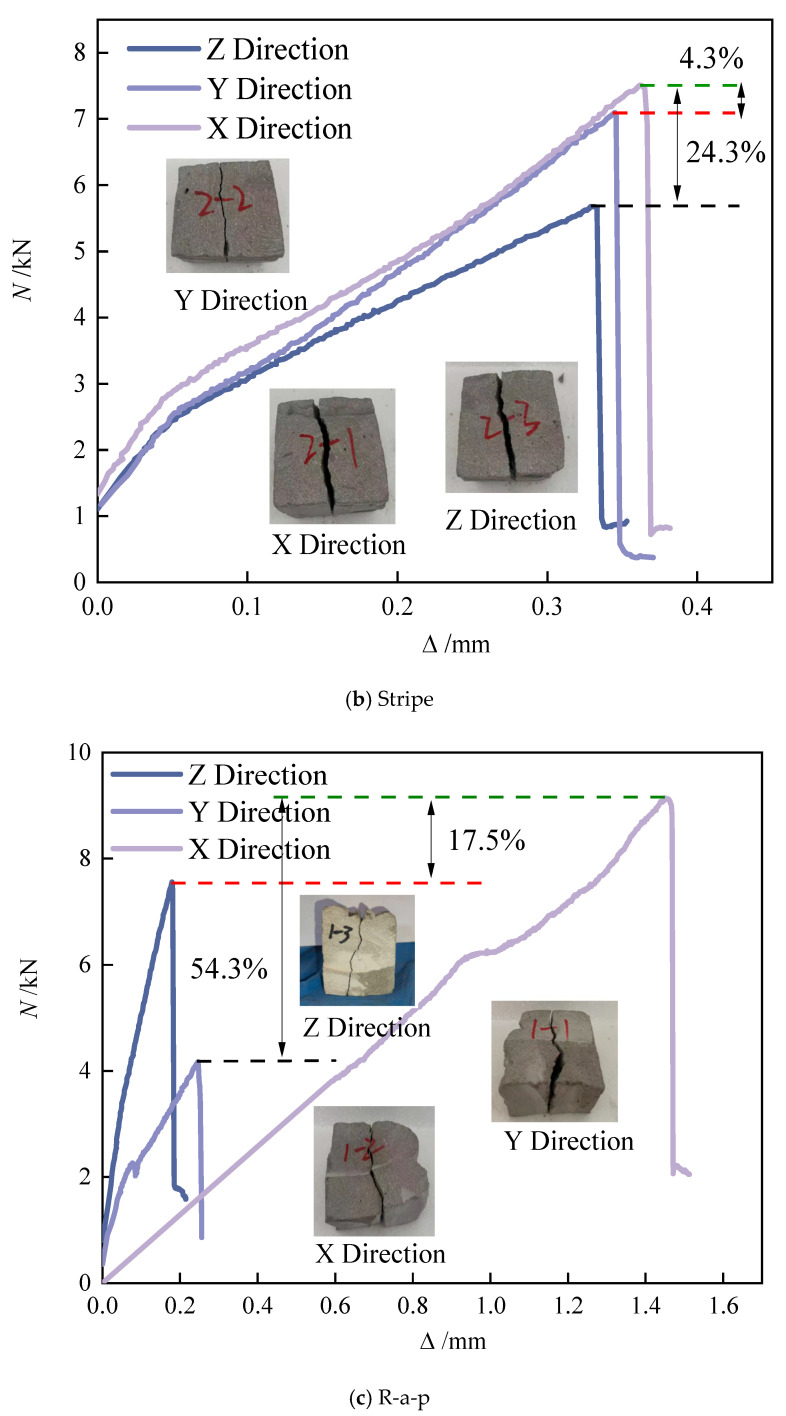
Load–displacement curves of the splitting test block under different loading directions.

As shown in Figure 8, all three splitting tensile load–displacement curves exhibit similar behavioral trends: an initially linear elastic response followed by abrupt post-peak softening after reaching maximum load. This indicates brittle fracture at critical loading, with failure planes propagating completely through the specimen core. Significant variations in load-bearing behaviors exist between specimen types. Among the specimens, Corner specimens exhibit the highest Z-direction load capacity with minimal displacement range, indicating rapid stress-concentrated failure along the principal loading axis. Stripe specimens demonstrate superior X-direction bearing capacity, with significantly enhanced displacement ductility compared to Corner specimens, suggesting that internal structural mechanisms (fiber pull-out or interface debonding) prolong failure progression. R-a-p specimens show 4.3% and 24.3% reductions in Y- and Z-direction capacities relative to the X-orientation, respectively. With maximum displacement limited to 0.38 mm—the lowest ductility among specimen types—R-a-p’s failure mode exhibits complex asymmetric fracture propagation attributed to multiaxial stress interactions. These performance variations originate from fundamental differences in printed geometry, material anisotropy, and loading directionality, demonstrating the structural design’s significant influence on splitting tensile behavior.

**Figure 9:**
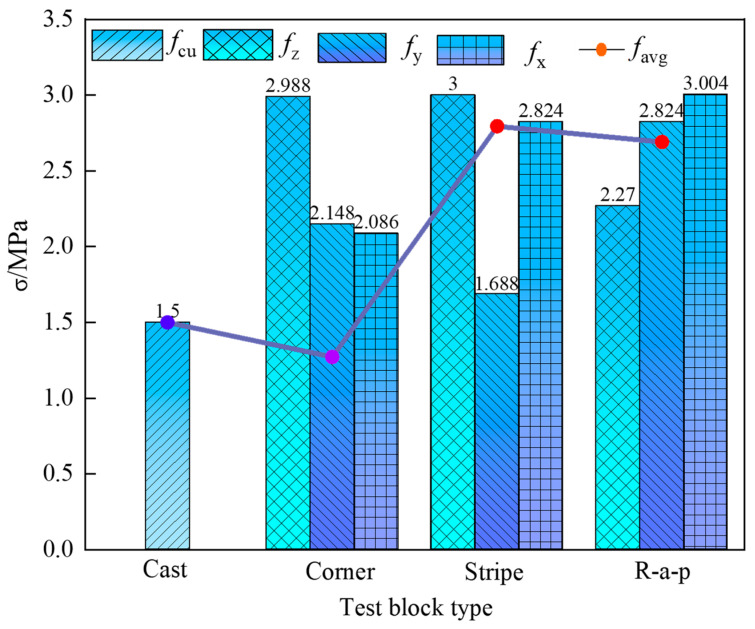
Comparison of the splitting strength of the three printed test blocks and the splitting strength of the cast-in-place test blocks.

Figure 9 illustrates the relationship between specimen configuration and splitting tensile strength distribution. Both Corner and Stripe specimens exhibit enhanced Z-direction tensile strength due to confinement effects generated by their geometric arrangement. In these specimens, the initially printed perimeter concrete restricts the lateral deformation of the inner striped regions, resulting in improved compaction and stronger interlayer bonding. This confinement effect directly enhances tensile performance, with Corner specimens achieving the highest Z-direction strength of 2.988 MPa, indicating a concentrated stress path that is aligned with the principal loading axis. This correlates with the sharp brittle fracture behavior observed in the load–displacement curves.

In Stripe specimens, the geometric pattern introduces discontinuities and misalignment in the X-direction, disrupting stress transfer. Consequently, their tensile strength in the X-direction (1.688 MPa) is significantly lower than in the Z-direction (3 MPa), as shown in Table 4. The irregular geometric path alters internal stress trajectories, leading to multidirectional failure coupling.

R-a-p specimens show slightly lower Z-direction strength compared to Corner specimens, but their overall mean tensile strength surpasses that of the Corner specimens by 52.96%. This improvement could be due to preferential fiber orientation or enhanced interfacial bonding mechanisms in the R-a-p specimens. Interestingly, the X-direction strength of R-a-p specimens remains higher than the Z-direction strength, which may be attributed to lateral constraints or geometric asymmetry within the specimen’s structure.

In general, all specimens exhibit superior Z-direction strength, supporting the design premise that the Z-axis should dominate the load-bearing capacity. However, the anomaly observed in R-a-p specimens, where the X-direction strength exceeds that of the Z-orientation, emphasizes the critical role that lateral constraints or non-uniform geometric properties can play in the tensile performance. Overall, the splitting tensile strength of 3D-printed concrete is strongly influenced by the geometric configuration, material anisotropy, and loading directionality, as evidenced by the performance variations among specimen types [33].

### 3.3. Analysis of DIC Test Results

**Figure 10:**
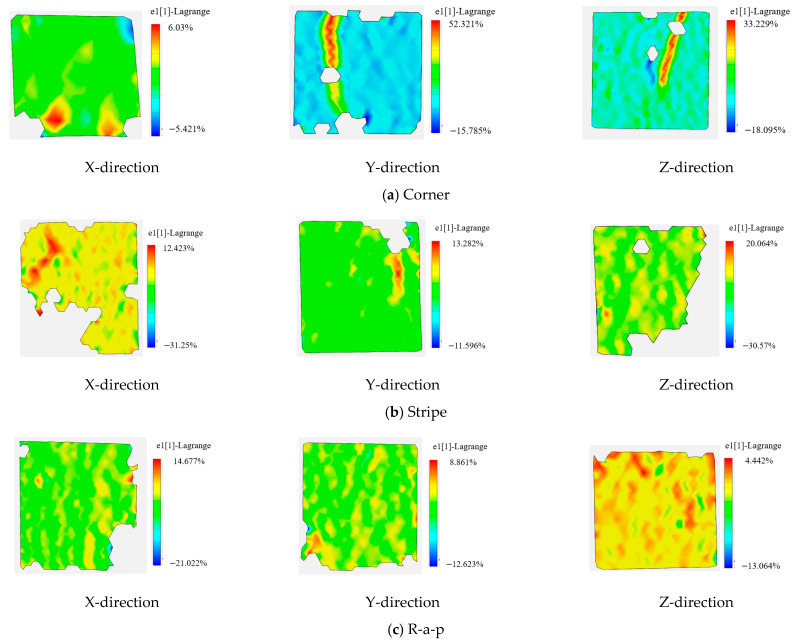
DIC-based first principal strain field (e_1_) during failure of the 3D-printed specimen, showing localized strain concentration along interfacial zones.

**Figure 11:**
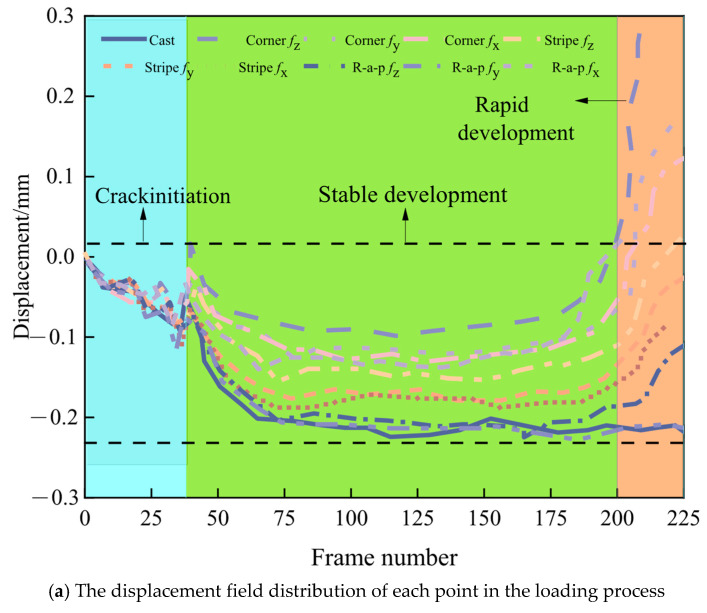

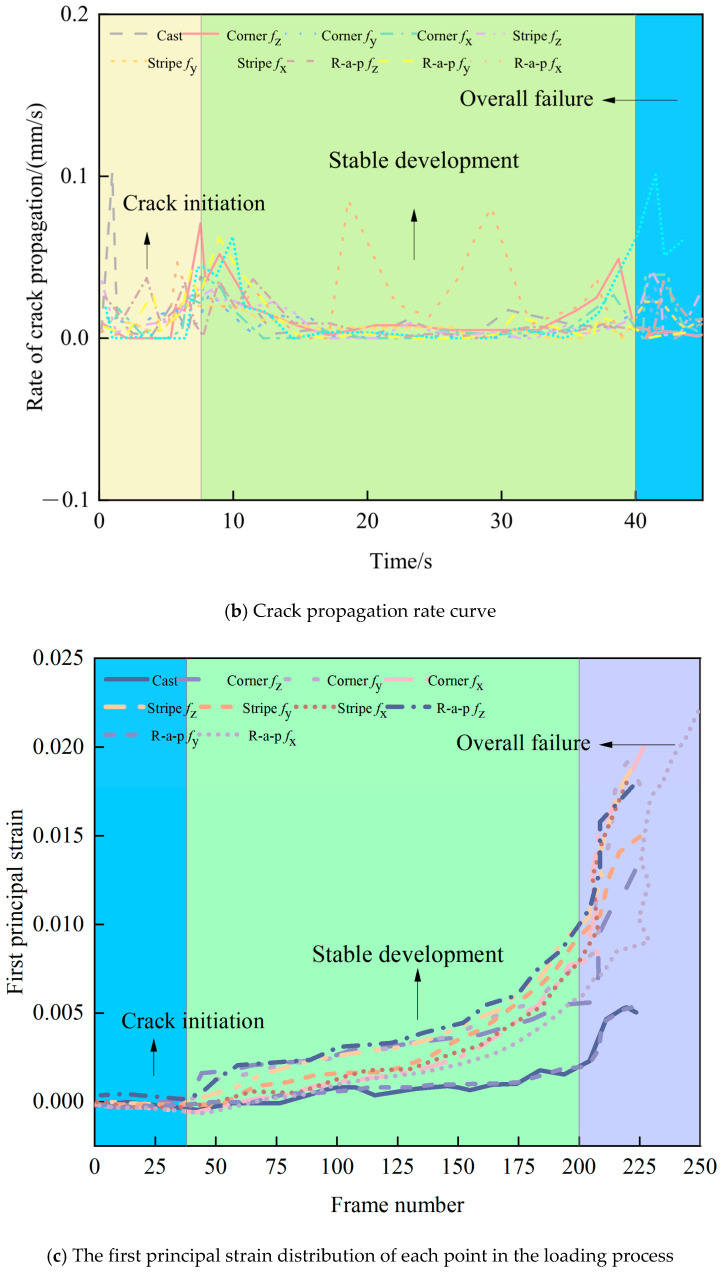
The crack length, propagation rate, and the first principal strain data of different types of test blocks under compression are shown.

As shown in Figure 10 and Figure 11, cracks in cast specimens propagate in near-vertical orientations. Following primary crack formation, secondary cracks develop progressively until ultimate failure.

During fracture progression, displacement measurements along crack paths exhibit triphasic behavior: an initial increase and a subsequent decrease, followed by renewed increases with fluctuating progression. The first principal strain remains relatively constant during initial loading but undergoes an abrupt change at t = 48 s, indicating rapid crack tip advancement and progressive energy release, culminating in specimen failure [34].

When the 3D-printed concrete cube specimen was loaded along the x-direction, cracks initially appeared at the specimen’s edge. As loading progressed, these cracks propagated both upwards and downwards, ultimately leading to the crushing of the cube. A significant point in the loading process occurred at 8 s. Prior to 8 s, the displacement field exhibited fluctuations. After 8 s, displacement increased slowly, followed by a sharp increase at 10 s. Subsequently, the first principal strain increased sharply, accompanied by rapid crack tip propagation. The specimen completely lost its load-bearing capacity following the formation of both primary and secondary cracks.

In a separate test under x-direction loading, crack propagation began at the edge and extended gradually from the bottom to the top. Before 4 s, displacement generally increased and then stabilized. At 5 s, displacement began to fluctuate. An analysis of the first principal strain indicated a sharp expansion of the crack tip at approximately 5 s. Following this, the first principal strain increased steadily until the specimen failed.

When the 3D-printed concrete cube specimen was loaded along the x-direction, cracks propagated roughly along a 45-degree diagonal plane, ultimately leading to rupture. The displacement at measurement points initially exhibited a fluctuating increase, followed by a stabilization period and then a continued rising trend.

The first principal strain underwent a significant/dramatic change at approximately 50 s, subsequently displaying a fluctuating increase. This behavior indicates that strain energy release commenced at around 10 s, accompanied by gradual crack tip propagation until the specimen failed.

**Figure 12:**
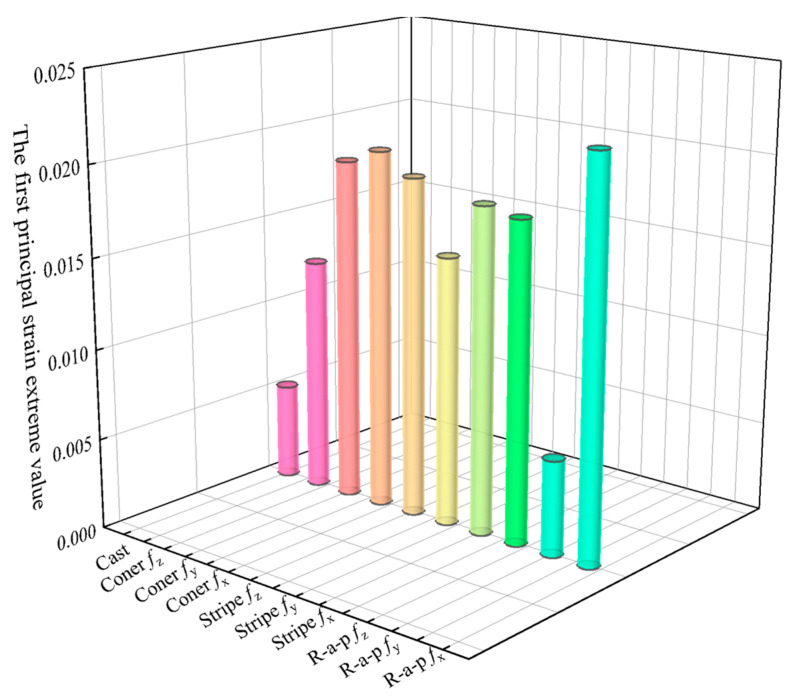
The first principal strain data of different types of test blocks under compression.

Under compressive loading, 3D-printed concrete specimens exhibit markedly distinct mechanical behavior compared to cast specimens, as shown in Figure 12. The average first principal strain for traditionally cast concrete is 0.00532, whereas 3D-printed specimens display average values ranging from 0.01503 to 0.01737, demonstrating pronounced anisotropy.

The directional dependence analysis reveals that the Corner specimen exhibits the highest stiffness in the Z-direction. Conversely, Stripe and R-a-p specimens show weaker stiffness, indicating process-dependent variations in interlayer bonding performance. Notably, the Y-direction strain in the R-a-p specimen is comparable to that of traditional cast concrete, suggesting that specific printing paths can optimize the material toward near-isotropic behavior.

Although basalt fiber reinforcement enhances overall toughness, its preferential alignment exacerbates anisotropy. This effect is particularly evident in the R-a-p specimen, where X-direction strains reach 0.02199—a value predisposing the material to interfacial deformation dominance at layer boundaries. These findings indicate that balancing toughening effects with anisotropy control requires the coordinated regulation of fiber distribution and interlayer bonding during the printing process.

### 3.4. Analysis of CT Test Results

**Figure 13:**
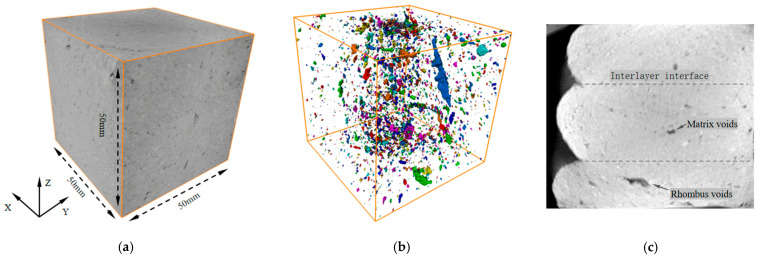
CT slices and three-dimensional reconstruction of 3D-printed concrete patterns. (**a**) Three-dimensional reconstruction of the Corner test block. (**b**) Corner test block pore’s three-dimensional map. (**c**) CT slices of the Corner test block.

Figure 13 presents the 3D reconstruction model of a printed corner concrete specimen and its corresponding CT image slice in the z-plane, revealing the distribution of meso-scale defects within the material. The CT images show that variations in grayscale intensity reflect differences in material density, with darker regions corresponding to low-density features such as air voids and water-filled pores, while gray areas represent a cementitious matrix [35]. The reconstruction model identifies four characteristic defect types: interlayer microvoids, which are formed between adjacent printed layers and are typically less than 100 μm in size and concentrated at vertical interlayer interfaces; elongated interfacial voids, which are diamond-shaped cavities formed between horizontal printed filaments, with a width greater than 100 μm and a length of less than or equal to 10 mm, resulting from variations in filament width; matrix pores, which are intrinsic microdefects from cement hydration and mixing processes and are retained due to the absence of vibration compaction; and filament interfacial transition zones (ITZs), which are weak interfacial zones formed by extrudate water films that significantly degrade bond performance. In contrast, pores in conventionally cast concrete specimens predominantly exhibit near-spherical morphology with low internal irregularity [36].

**Figure 14:**
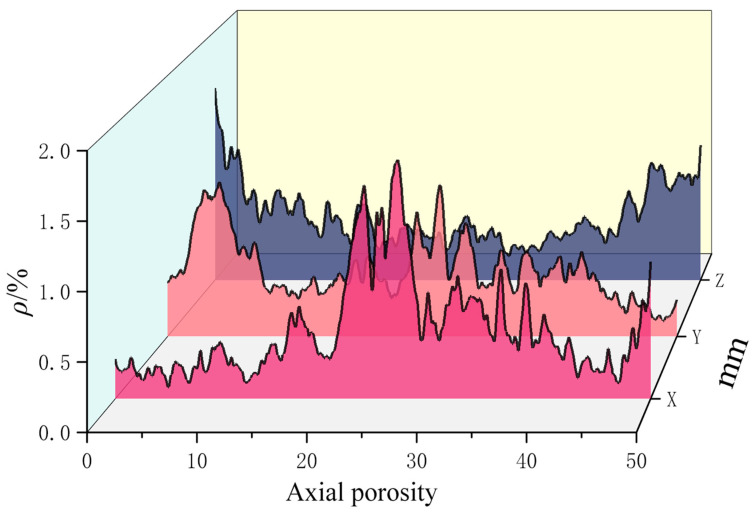
Porosity distribution of different axial surfaces.

As shown in Figure 14, the spatial differentiation of defects is revealed through porosity distribution profiles along the X, Y, and Z axes:

X-Direction (Parallel to Filament Axis): Peak porosity (1.73%) occurs at positions 20–28 mm, attributed to the cumulative effects of macroscopic elongated diamond-shaped cavities. The lower porosity range (0.08–0.67%) at positions 0–20 mm reflects uniformly distributed matrix micropores.

Y-Direction (Perpendicular to Interlayer Planes): Porosity increases sharply to 1.18% within 0–5 mm due to the dense clustering of interlayer micropores, confirming spatial coincidence between interlayer weak interfaces and interfacial transition zones.

Z-Direction (Build Direction): Porosity exhibits an initial decrease followed by an increase, peaking at 1.545% (0–1 mm position). This profile reveals the size-specific concentration of defects (100–500). 

Extrusion-induced shear rheology results in water film retention on the outer filament surface, leading to weak interfacial bonding zones. The measured porosity range of 0.191% to 1.545% within the 0–50 mm section confirms bubble entrapment due to the absence of vibration compaction.

The correlation analysis reveals strong process–parameter dependence in directional pore distributions, as shown in Table 5:

In the X-direction, elongated voids are primarily controlled by extrusion stability. Y-direction interlayer defects are dependent on layer-height precision. The porosity within the Z-direction interfacial transition zones is influenced by rheological properties and the water–cement ratio. High-porosity regions serve as preferential pathways for crack propagation.

Extrusion-induced shear rheology results in water film retention on the outer surface of the filament, leading to weak interfacial bonding zones. The measured porosity, ranging from 0.191% to 1.545% in the 0–50 mm section, confirms the entrapment of air bubbles, which occurs due to the absence of vibration compaction during the printing process.

Correlation analysis reveals strong process–parameter dependence in directional pore distribution.

The X-direction elongated voids are extrusion-stability-controlled. The Y-direction interlayer defects are layer-height-precision-dependent. The Z-direction interfacial transition zone’s porosity correlates with rheological properties and the water–cement ratio. High-porosity regions constitute preferential propagation pathways for crack advancement.

### 3.5. Microstructure Analysis

**Figure 15:**
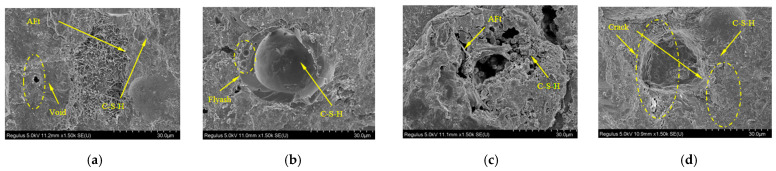
SEM diagram of 3D-printed concrete: (**a**) 3D-printed concrete crystal precipitation zone’s SEM diagram; (**b**) SEM image of the C-S-H enrichment zone of 3D-printed concrete; (**c**) 3D-printed coagulation pore area SEM diagram; (**d**) SEM images of the 3D-printed concrete interface area.

The microstructure of 3D-printed calcium sulfoaluminate (CSA) cement concrete fundamentally governs its mechanical properties. Its primary hydration product—acicular ettringite (AFt) crystals—forms an intricate three-dimensional ‘bird’s nest’ skeletal structure under SEM observation [37] (Figure 15). This framework rapidly develops within 1–3 h, effectively filling interlayer pores. Compared to conventional basalt fiber-reinforced concrete, CSA specimens exhibit denser crystalline frameworks and lower initial porosity.

Furthermore, the hydration of high-alumina phases in CSA cement releases substantial Ca^2+^ and Al^3+^ ions, promoting preferential AFt nucleation and growth at interlayer interfaces. This creates crystalline bridging structures that enhance interfacial bonding. Rapid hydration enables the partial penetration of freshly deposited paste into underlying layers under gravitational forces, with subsequent AFt whisker development further strengthening interfaces through mechanical interlocking.

While printing introduces flattened interlayer pores, AFt’s expansive nature compresses pore boundaries, resulting in AFt crystal adhesion along pore walls. Notably, CSA systems demonstrate lower autogenous shrinkage and reduced crack widths compared to ordinary Portland cement. SEM micrographs reveal microcracks being ‘sutured’ by AFt crystals, with crack interiors filled by AFt bundles [32]. Regarding mechanical performance, interlayer bonding failure constitutes the primary failure mechanism. Although AFt improves interfacial bonding, crystallographic orientation differences in the skeletal structure at interlayer interfaces promote crystal fracture over matrix failure under Z-direction loading.

Furthermore, interlayer pores initiate microcrack propagation at stress concentration points. These cracks extend along pore peripheries and traverse multiple AFt crystals, exhibiting characteristic brittle fracture behavior.

Correlative SEM/CT analyses quantify the spatial differentiation of multiscale defects.

Spatial coincidence between flattened interlayer pores and Z-direction porosity peaks confirms that AFt expansion only locally compresses voids. Elongated voids with continuous distributions of >100 μm in the X-direction correlate precisely with crystalline-bridging fracture paths in SEM images.

This demonstrates that process-induced defects dominate crack propagation directionality. Material performance results from the coupled mechanisms of rapid AFt formation and printing rheology, necessitating multiscale synergistic strengthening through controlled AFt content and graded interfacial reinforcement at weak interlayer planes.

### 3.6. Finite Element Model Verification and Mechanism Analysis of 3D Printed Concrete

**Figure 16:**
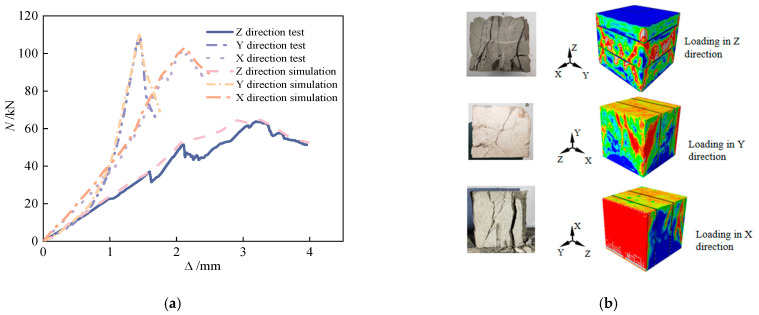
Compression block test and simulation failure mode comparison. (**a**) Compression test and load–displacement curve simulation comparison. (**b**) Comparison of compression test and simulated failure modes.

**Figure 17:**
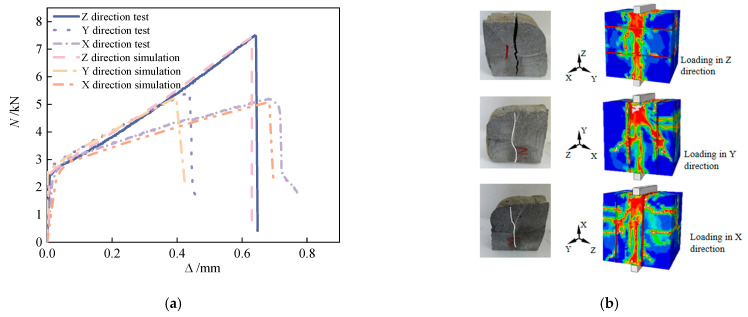
Comparison of splitting block test and simulated failure modes. (**a**) Comparison of splitting test and simulated load–displacement curves. (**b**) Comparison of splitting test and simulated failure modes.

The forces corresponding to the formation of cracks and ultimate failure, based on both experimental tests and FEM simulations, are detailed in Table 6. Figure 16 and Figure 17 illustrate the pronounced anisotropic failure behavior observed in 3D-printed concrete specimens under compressive and splitting tensile loading, respectively. These figures form the core of this study by revealing both the directional dependence of damage patterns and the correlation between experimental and numerical results.

To further interpret the damage mechanisms, it is important to highlight both the concordances and discrepancies between experimental observations and simulation results. In the compression tests, for example, cracks in the X-direction primarily propagate along filament interfaces, while Y- and Z-direction cracks typically follow interlayer planes—consistent with the inherent anisotropic structure of 3D-printed materials. In simulations, these failure paths were effectively replicated by incorporating anisotropic weakening at the filament and interlayer interfaces, demonstrating strong agreement.

However, slight deviations were noted, particularly in the Z-direction splitting simulations, where simulated cracks exhibited more idealized propagation paths compared to the somewhat irregular stepwise or branching cracks observed experimentally. This mismatch can be attributed to local heterogeneities, pore-induced stress concentrations, or interface geometry complexity that is not fully captured by the simplified interface modeling in the FEM environment.

Despite these minor inconsistencies, the simulations show excellent overall fidelity. The relative error in peak load, elastic modulus, and softening curve progression remained within 9.7%, confirming the model’s predictive capability. Thus, Figure 15 and Figure 16 not only reinforce the critical role of material anisotropy induced by the printing process but also underline the need for enhanced interface modeling to bridge local-scale deviations in future work.

**Figure 18:**
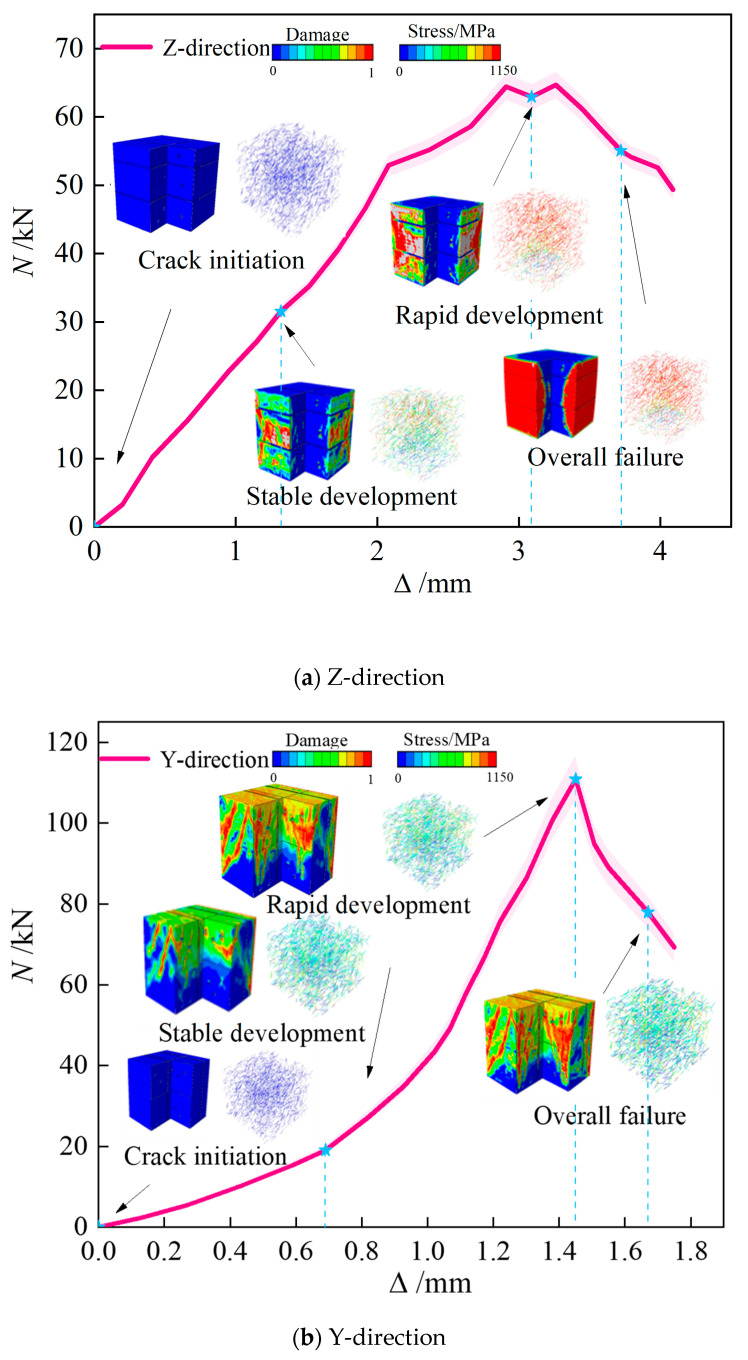

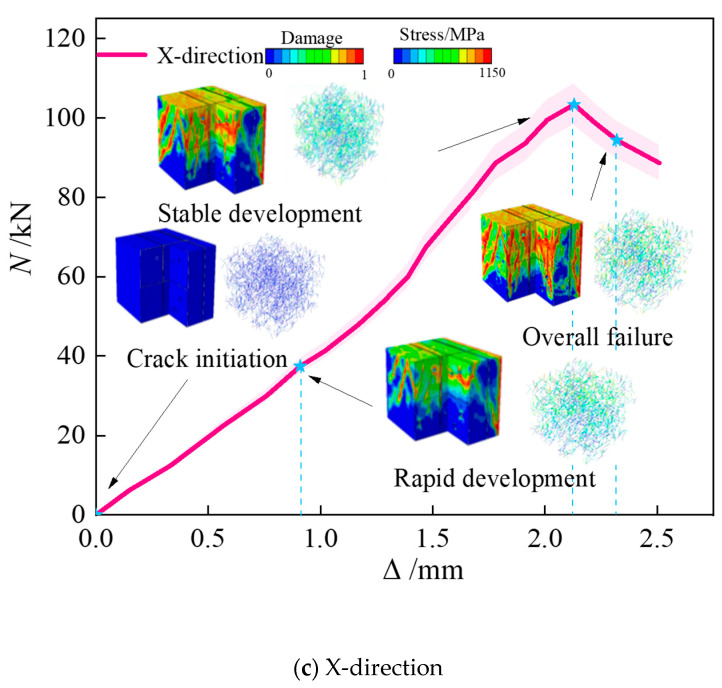
Failure process of 3D-printed concrete compression test block under different directions.

As shown in Figure 18a, the Corner specimen exhibits the most pronounced anisotropy along the build direction (Z-axis). Elastic Stage: Weak interlayer interfaces dominate mechanical response, with interfacial bond strength measuring only 30–40% of the matrix’s strength. During printing, fibers align predominantly within the XY-plane, resulting in sparse fiber density along the Z-direction. This fiber orientation deficiency prevents effective fiber bridging across interlayers, enabling rapid interfacial slip and stress concentration development. Elastoplastic Stage: Damage propagates rapidly along interlayer planes. The longitudinal fiber distribution provides insufficient constraint against through-thickness debonding. Finite element simulations confirm that primary damage concentrates at interfacial debonding sites, while matrix pores initiate microcracks. Plastic Stage: Complete interlayer debonding triggers progressive layer collapse. Minimal fiber pull-out resistance in the Z-direction shifts energy dissipation primarily to matrix failure mechanisms, manifested as post-peak softening in the load–displacement response.

As shown in Figure 18b, failure mechanisms perpendicular to the filament alignment direction exhibit tension–shear coupling characteristics. Elastic Stage: Loading perpendicular to inter-filament interfaces generates normal tensile stresses that concentrate within weak planes. Fiber alignment along printing paths restricts bridging capability in the Y-direction, reducing the interfacial tensile strength to 50–60% of the matrix value. Elastoplastic Stage: Increasing interfacial tensile stresses cause fiber reorientation under transverse tension. Partial fiber–matrix debonding nucleates microcracks, which propagate along inter-filament weak bands through interface-dominated damage. Plastic Stage: Interface coalescence triggers 45° diagonal cracks. Fiber pull-out mechanisms decelerate shear displacement rates, enhancing residual strength by approximately 20%. However, inter-filament dislocation ultimately causes shear–splitting failure. Optimizing fiber content enhances interfacial bonding and suppresses premature failure initiation.

As shown in Figure 18c, the specimens exhibit optimal mechanical performance along the filament alignment direction (X-axis), demonstrating high synergy between fiber reinforcement and printing path. Elastic Stage: Stress distributes uniformly along continuous filaments. X-aligned fibers significantly enhance matrix stiffness, increasing elastic modulus by 15–20% compared to other orientations. Interfacial shear stresses remain negligible. Elastoplastic Stage: Matrix microcracks are constrained by fiber bridging, resulting in tortuous, bifurcated propagation paths. Fiber–matrix mechanical interlocking delays interfacial slip, causing multi-stage stiffness degradation. Plastic Stage: Failure couples matrix crushing with interfacial slip. Fiber pull-out energy dissipation mechanisms increase residual strength by >30%, producing a characteristic multi-stage softening plateau.

**Figure 19:**
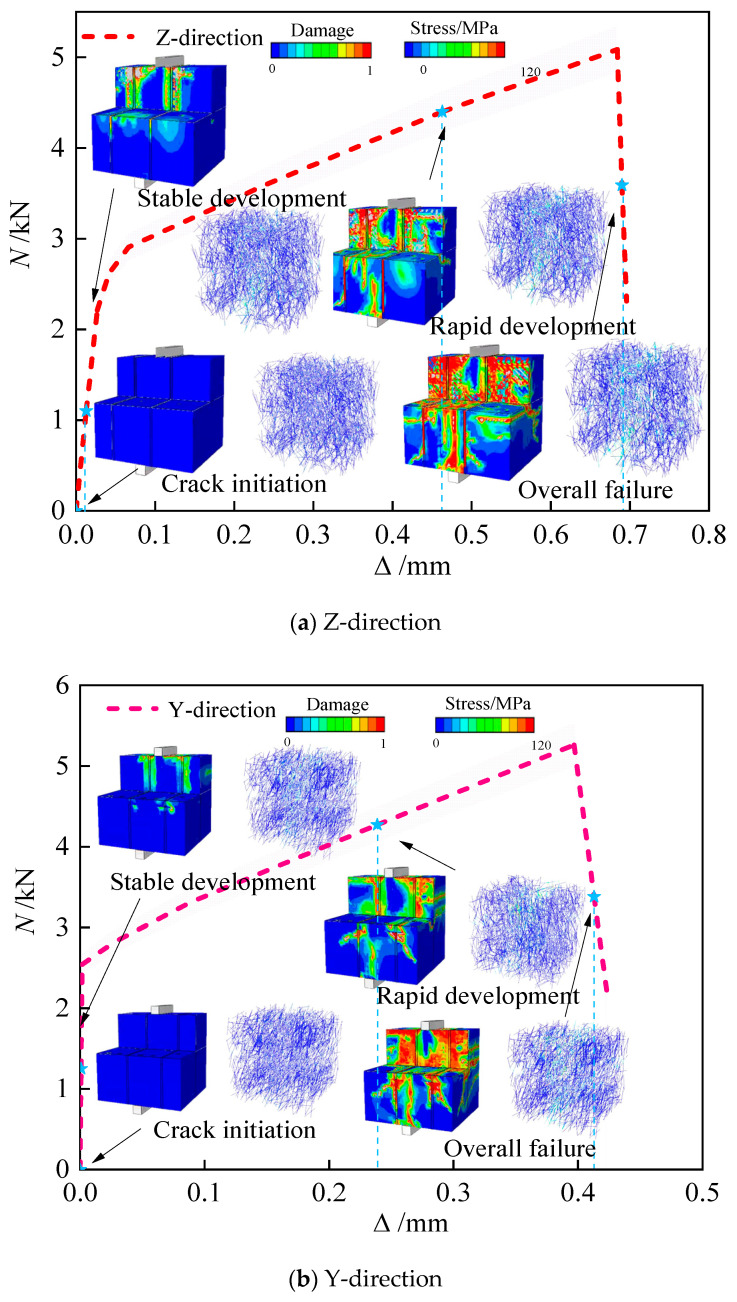

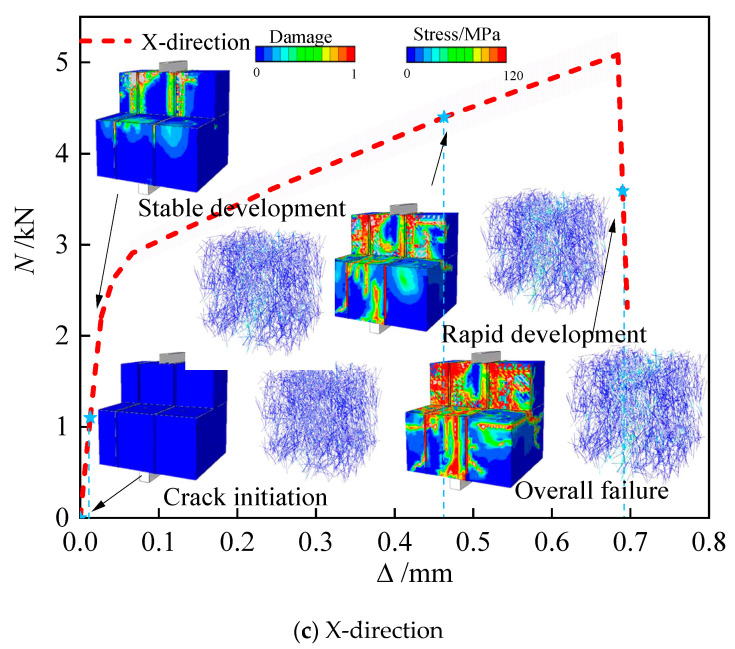
The failure process of the 3D-printed concrete splitting test block in different directions.

As shown in Figure 19a, in the Elastic Stage, damage contours indicate uniform stress distribution within the upper matrix, with no interlayer stress concentration. Transversely oriented fibers remain in a low-stress state. Elastoplastic Stage: An orange damage zone emerges at interlayer interfaces, signaling incipient bond failure. Due to discontinuous fiber distribution across layers, stress concentration develops in vertically aligned fibers beneath the loading platen, limiting their bridging effectiveness. Plastic Stage: A red damage zone propagates horizontally along interlayer planes. Fiber stress contours confirm the fracture of interfacial fibers, resulting in complete interlayer debonding that produces a planar fracture surface–indicative of characteristic brittle failure behavior.

As illustrated in Figure 19b, in the Elastic Stage, the damaged nephogram appears to be uniformly blue. Continuous highlighted bands, corresponding to fiber bundles aligned along the printing paths, are displayed in the stress nephogram, with axial stresses exceeding 60 MPa. Elasto-Plastic Stage: An orange damaged zone emerges at the tip of the longitudinal crack. However, the fiber stress nephogram indicates that the stress in the fibers at the crack location increases to 80 MPa. Plastic Stage: Damage propagates along the interfacial transition zone (ITZ), manifesting as slender red band-shaped failure (likely interfacial debonding/sliding) and vertical tensile failure. The fiber stress nephogram records an increase in stress from 80 MPa to 120 MPa. This stress escalation corresponds to the progressive pull-out process of the fibers. The fracture surface exhibits failure characterized by fiber protrusion.

As depicted in Figure 19c, in the Elastic Stage, the damaged nephogram reveals slight damage initiation within the interlaced regions between layers. Concurrently, the longitudinal fiber stress nephogram exhibits continuous high-stress zones. Elasto-Plastic Stage: Cracks propagate alternately along the interlayer interfaces and transverse fiber bands. The damage nephogram displays bifurcating orange paths indicative of damage propagation. Within the stress nephogram, the inclined fibers are observed to reach their critical stress first. Plastic Stage: Multiple cracks coalesce, forming a mesh-like damage band. The transverse fiber stress nephogram indicates that fibers beneath the loading platen undergo shear-induced debonding failure.

## 4. Conclusions

This study investigates the mechanical properties and failure mechanisms of composite 3D-printed concrete using compressive and splitting tensile tests, CT scanning, SEM microstructural analysis, and three-dimensional mesoscale numerical simulations. The following conclusions were drawn:

1. The compressive strength of composite basalt fiber 3D-printed concrete exhibits pronounced anisotropy. Among the specimens, the Corner-type sample showed the highest compressive strength in the X direction (43.6 MPa), which is approximately 18% lower than that of cast concrete. The R-a-p specimen exhibited the lowest compressive strength in the Z direction, showing a reduction of up to 42% compared to cast concrete. Anisotropy coefficients indicate that the R-a-p specimen displays the most significant anisotropy (*I*a = 31.22), while the Corner specimen shows the least significant anisotropy (*I*a = 17.08).

2. Different geometric configurations of the specimens result in distinct crack initiation points, propagation paths, and ultimate failure modes. The Stripe specimen exhibited complex stress paths, with a tensile strength of 1.688 MPa in the Y direction, significantly lower than 2.824 MPa in the Z direction. This suggests that its irregular printing path induces multi-directional coupled failure. In contrast, the Cast specimen showed nearly vertical crack propagation, indicative of a typical global failure pattern.

3. Due to a more uniform fiber orientation, the R-a-p specimen benefits from enhanced bridging and anchoring effects. Its average splitting tensile strength reached 3.39 MPa, representing a 52.96% increase compared to the Corner specimen. During loading, strain at the crack tip was delayed, and a sharp change in the displacement curve occurred after 10 s. The interaction of primary and secondary cracks significantly postponed the failure process.

4. Digital Image Correlation (DIC) tests reveal that during crack propagation, principal strain experiences sudden shifts accompanied by rapid release of strain energy. For the cast specimen, a sudden increase in principal strain occurred at 48 s, indicating accelerated crack tip growth. In 3D-printed specimens, particularly those with diagonal crack patterns, strain surges appeared at around 50 s, followed by fluctuating increases—demonstrating strong coupling between crack propagation and energy release.

The splitting performance of 3D-printed concrete is jointly influenced by geometric configuration, loading direction, and fiber reinforcement mechanisms. The rational optimization of printing paths and fiber orientation can further improve structural performance and fracture toughness. The findings provide theoretical insights and data support for the configuration optimization, crack resistance design, and performance prediction of 3D-printed concrete structures under multi-directional loading, offering significant value for engineering applications.

In addition to these theoretical contributions, the practical implications for real-world 3D printing in construction are considerable. The optimization of printing paths, based on the observed porosity and mechanical performance characteristics, can be used to enhance the mechanical properties of 3D-printed concrete in critical regions, improving overall structural integrity. Furthermore, this study emphasizes the role of material composition and fiber reinforcement in controlling porosity levels and mechanical properties. By adjusting mix design parameters, such as the water–cement ratio and fiber content, the performance of 3D-printed concrete can be optimized for different loading conditions, making it a more durable and cost-effective material for large-scale construction projects. These insights offer valuable support for future work in print path optimization and material design, ensuring the continued development and application of 3D-printed concrete in the construction industry.

However, challenges remain, and the pathway forward is not without controversy. One pressing issue is whether performance enhancement should focus more on advanced material modifications (e.g., nanomaterials, rheology control agents) or printing process interventions (e.g., real-time feedback for deposition geometry and layer compaction). Similarly, there is ongoing debate on whether passive fiber reinforcement is sufficient for suppressing anisotropic cracking or whether active reinforcement techniques, such as embedded rebar or hybrid reinforcement systems, should be integrated despite added complexity. Another open question is the applicability of lab-scale optimization strategies under on-site environmental conditions, where humidity, temperature, and equipment variability may limit repeatability. These controversial aspects present opportunities for future exploration and innovation, pushing the boundaries of what 3D-printed concrete can achieve in real-world applications.

## Figures and Tables

**Table 1:** Material quality ratio of 3D-printed concrete (%).

Portland Cement	Sulphoaluminate Cement	Silica Fume	Cellulose Ether	Basalt Fiber	River Sand	Water
36.73	2.04	2.04	0.02	0.04	40.72	18.37

**Table 2:** Summary of parameters for CDP constitutive model.

Parameter	Value/Description
Elastic Modulus (*E*)	32.5 GPa
Poisson’s Ratio (*ν*)	0.2
Compressive Strength (*f*_c_)	49.1 MPa
Tensile Strength (*f*_t_)	4.0 MPa
Damage Initiation Criteria	Maximum principal strain
Damage Evolution Law	Based on the degradation of stiffness as a function of accumulated damage
Fracture Energy	Model-based, dependent on material properties and loading conditions
Plastic Hardening	Associated with the yield function and damage evolution law in CDP

**Table 3:** Compressive mechanical properties and anisotropy coefficients of different types of blocks.

Test Block Type	Compressive Strength/MPa			Coefficient of Anisotropy
	Z	X	Y	
Cast	31			0
Corner	25.6	43.6	41.2	17.08
Stripe	23.2	7.76	5.84	35.12
R-a-p	9.2	15.6	14.8	31.22

**Table 4:** Splitting mechanical properties and anisotropy coefficients of different types of blocks.

Test Block Type	Cleavage Strength/MPa			Coefficient of Anisotropy
	Z	X	Y	
Cast	1.5			0
Corner	2.988	2.148	2.085	17.08
Stripe	3	1.688	2.824	35.12
R-a-p	2.27	2.824	3.004	31.22

**Table 5:** Summary ofporosity and mechanical properties for Corner specimen.

Direction	Porosity (%)	Compressive Strength (MPa)	Splitting Tensile Strength (MPa)
Z	0.191	25.6	2.988
Y	0.161	43.6	2.148
X	0.154	41.2	2.086

**Table 6:** Forces leading to crack formation and failure in different test blocks.

Test Block Type	Compressive Loading/kN			Splitting Load/kN		
	Z	X	Y	Z	X	Y
Cast	77.5			1.5		
Corner	64	109	103	7.47	5.37	5.21
Stripe	58	19.4	14.6	7.5	4.22	4.06
R-a-p	23	39	37	5.68	7.06	7.51

## Data Availability

The original contributions presented in this study are included in the article. Further inquiries can be directed to the corresponding author.

## References

[1] Gunasti A., Manggala A.S. (2024). Utilization of bamboo for concrete columns in earthquake-resistant simple houses in Indonesia. Case Stud. Constr. Mater..

[2] Alothman A., Mangalathu S., Al-Mosawe A., Alam M., Allawi A. (2023). The influence of earthquake characteristics on the seismic performance of reinforced concrete buildings in Australia with varying heights. J. Build. Eng..

[3] Harati M., van de Lindt J.W. (2024). Impact of long-duration earthquakes on successive earthquake-tsunami fragilities for reinforced concrete frame archetypes. J. Struct. Eng..

[4] Nodehi M., Aguayo F., Nodehi S.E., Gholampour A., Ozbakkaloglu T., Gencel O. (2022). Durability properties of 3D printed concrete (3DPC). Autom. Constr..

[5] Khan M., McNally C. (2024). Recent developments on low carbon 3D printing concrete: Revolutionizing construction through innovative technology. Clean. Mater..

[6] Garcés G., García-Alvarado R., Bunster V., Muñoz-Sanguinetti C. (2025). Additive Construction 4.0: A systematic review of 3D concrete printing for Construction 4.0. Eng. Constr. Archit. Manag..

[7] GivKashi R.M., Moodi F., Ramezanianpour M.A. (2025). Investigating shrinkage and mechanical properties of 3D-printed concretes under different curing conditions. Int. J. Civ. Eng..

[8] Zeng J.-J., Sun H.-Q., Deng R.-B., Yan Z.-T., Zhuge Y. (2025). Bond performance between FRP bars and 3D-printed high-performance concrete. Structures.

[9] Wang Q., Yang W., Wang L., Bai G., Ma G. (2025). Reinforcement design and structural performance for topology-optimized 3D-printed concrete truss beams. Eng. Struct..

[10] Zhao H., Wang X., Sun J., Wu F., Liu X., Chen Z., Wang Y. (2025). Automated analysis system for micro-defects in 3D-printed concrete. Autom. Constr..

[11] Raza S., Triantafyllidis Z., Anton A., Dillenburger B., Shahverdi M. (2024). Seismic performance of Fe-SMA prestressed segmental bridge columns with 3D-printed permanent concrete formwork. Eng. Struct..

[12] Liu C., Zhang R., Liu H., He C., Wang Y., Wu Y., Liu S., Song L., Zuo F. (2022). Analysis of mechanical performance and damage mechanism for 3D-printed concrete based on pore structure. Constr. Build. Mater..

[13] Wu Y., Liu C., Liu H., Bai G., Meng Y., Ding S. (2024). Mechanism of the influence of multiscale pore structure on triaxial mechanical properties of 3D-printed concrete with recycled sand. Cem. Concr. Compos..

[14] Wang H., Shao J., Zhang J., Zou D., Sun X. (2022). Bond-shear performances and constitutive model of interfaces between vertical and horizontal filaments of 3D-printed concrete. Constr. Build. Mater..

[15] Zhao Y., Li J., Zhao Z., Tang Q., Zhao J., Liu J. (2025). Damage mechanism of hollow-sandwich packed columns in basalt-fiber 3D-printed concrete. J. Liaoning Univ. Sci. Technol..

[16] Li B., Li K., Lyu X., Zhao C., Guan X. (2024). Microscopic mechanism and predictive calculation of mechanical properties of basalt-fiber modified 3D-printed cement-based materials. Case Stud. Constr. Mater..

[17] Xiao J., Liu H., Ding T. (2021). Finite-element analysis on the anisotropic behavior of 3D-printed concrete under compression and flexure. Addit. Manuf..

[18] Zhang Y., Zhu X., Li M., Zhang C., Zhang Y., Du X., Banthia N., Mechtcherine V., Carstensen J.V., Monteiro P.J.M. (2025). 3D-printing technology in concrete construction. Nat. Rev. Clean Technol..

[19] Le T.T., Austin S.A., Lim S., Buswell R.A., Law R., Gibb A.G.F., Thorpe T. (2012). Hardened properties of high-performance printing concrete. Cem. Concr. Res..

[20] Bi M., Tran P., Xia L., Ma G., Xie Y.M. (2022). Topology optimization for 3D concrete printing with various manufacturing constraints. Addit. Manuf..

[21] Liu Z., Li M., Wong T.N., Tan M.J. (2024). Effects of pore properties on mechanical performance of 3D-printed concrete units: Experimental and numerical methods. J. Build. Eng..

[22] Jiang X., Li Y., Yang Z., Li Y., Xiong B. (2024). Harnessing path optimization to enhance the strength of 3D-printed concrete. Buildings.

[23] Wang C., Chen B., Vo T.L., Rezania M. (2023). Mechanical anisotropy, rheology and carbon footprint of 3D-printable concrete: A review. J. Build. Eng..

[24] China Concrete & Cement-Based Products Association (2022). Test Method for Basic Mechanical Properties of 3D-Printed Concrete.

[25] Ministry of Construction of the People’s Republic of China (2003). Standard for Test Methods for Mechanical Properties of Ordinary Concrete.

[26] Yao Y., Zhang J., Sun Y., Pi Y., Wang J., Lu C. (2024). Mechanical properties and failure mechanism of 3D-printed ultra-high-performance concrete. Constr. Build. Mater..

[27] Chen Y., Kuva J., Mohite A., Li Z., Rahier H., Al-Neshawy F., Shu J. (2023). Investigation of the internal structure of hardened 3D-printed concrete by X-CT scanning and its influence on mechanical performance. Materials.

[28] Sayyafzadeh B., Omidi A., Rasoolan I. (2019). Mesoscopic generation of random concrete structure using an equivalent space method. J. Soft Comput. Civ. Eng..

[29] Li F., Hu X., Shahzad Q. Anisotropic behavior in 3D-printed concrete: Finite-element simulation approach. J. Mater. Eng. Perf..

[30] Mader T., Schreter-Fleischhacker M., Shkundalova O., Neuner M., Hofstetter G. (2023). Constitutive modeling of orthotropic nonlinear mechanical behavior of hardened 3D-printed concrete. Acta Mech..

[31] Hassan B.R., Manguri A., Hussein A.B., Corrado A., Abdulrahman P.H., Mohammed L.M., Mahmood S.S., Hatim L.A. (2025). Experimental and numerical assessment of recycled plastic fibers on the shear strength and behavior of reinforced-concrete beams with basalt FRP bars. Eng. Struct..

[32] Xia Z., Geng J., Zhou Z., Liu G. (2025). Comparative analysis of polypropylene, basalt and steel fibers in 3D-printed concrete: Effects on flowability, printability, rheology and mechanical performance. Constr. Build. Mater..

[33] Hassan B.R., Yousif A.R. (2024). Experimental and analytical investigation of the shear behavior and strength of haunched beams reinforced with basalt-fiber-reinforced-polymer rebars. J. Bridge Eng..

[34] Moelich G.M., Kruger J., Combrinck R. (2020). Plastic shrinkage cracking in 3D-printed concrete. Compos. Part B-Eng..

[35] Liu C., Liu H., Wu Y., Wu J., Ding S. (2025). Effect of X-ray CT-characterized pore structure on freeze—thaw resistance of 3D-printed concrete with recycled coarse aggregate. Constr. Build. Mater..

[36] Zhang K., Lin W., Zhang Q., Wang D., Luo S. (2024). Evaluation of anisotropy and statistical parameters of compressive strength for 3D-printed concrete. Constr. Build. Mater..

[37] Yu S., Xia M., Sanjayan J., Yang L., Xiao J., Du H. (2021). Microstructural characterization of 3D-printed concrete. J. Build. Eng..

